# Use of Telemedicine Healthcare Systems in Children and Adolescents with Chronic Disease or in Transition Stages of Life: Consensus Document of the Italian Society of Telemedicine (SIT), of the Italian Society of Preventive and Social Pediatrics (SIPPS), of the Italian Society of Pediatric Primary Care (SICuPP), of the Italian Federation of Pediatric Doctors (FIMP) and of the Syndicate of Family Pediatrician Doctors (SIMPeF)

**DOI:** 10.3390/jpm13020235

**Published:** 2023-01-28

**Authors:** Susanna Esposito, Cristiano Rosafio, Francesco Antodaro, Alberto Argentiero, Marta Bassi, Paolo Becherucci, Fabio Bonsanto, Andrea Cagliero, Giulia Cannata, Fabio Capello, Fabio Cardinale, Tiziana Chiriaco, Alessandro Consolaro, Angelica Dessì, Giuseppe Di Mauro, Valentina Fainardi, Vassilios Fanos, Alfredo Guarino, Giada Li Calzi, Elisa Lodi, Mohamad Maghnie, Luca Manfredini, Emanuela Malorgio, Nicola Minuto, Maria Grazia Modena, Rossano Montori, Andrea Moscatelli, Elisa Patrone, Elena Pescio, Marco Poeta, Angelo Ravelli, Maddalena Spelta, Agnese Suppiej, Sergio Vai, Luca Villa, Rinaldo Zanini, Renato Botti, Antonio Vittorino Gaddi

**Affiliations:** 1 Pediatric Clinic, Department of Medicine and Surgery, University of Parma, 43126 Parma, Italy; 2 Family Pediatrics, AUSL Modena, 41124 Modena, Italy; 3 Department of Pediatrics, IRCCS Giannina Gaslini, 16147 Genoa, Italy; 4 Family Pediatrics, 50139 Florence, Italy; 5 Alma Mater University, 40126 Bologna, Italy; 6 Family Pediatrics, 10121 Turin, Italy; 7 UO Territorial Pediatrics, Primary Care Department, AUSL Bologna, 40126 Bologna, Italy; 8 UOC of Pediatrics and ED with an Allergy-Pneumological and Immuno-Rheumatological Focus, Giovanni XXIII Pediatric Hospital, University of Bari, 70124 Bari, Italy; 9 Health Department, ASL Roma 3, 00125 Rome, Italy; 10 General Management, IRCCS Istituto Giannina Gaslini, 16147 Genoa, Italy; 11 Pediatric and Rheumatology Clinic, IRCCS Istituto Giannina Gaslini, 16147 Genoa, Italy; 12 Department of Neurosciences, Rehabilitation, Ophthalmology, Genetics and Maternal-Child Sciences (DINOGMI), University of Genoa, 16126 Genoa, Italy; 13 Department of Surgical Sciences, University of Cagliari, 09127 Cagliari, Italy; 14 Family Pediatrics, 81100 Caserta, Italy; 15 Section of Pediatrics, Department of Translational Medical Sciences, University of Naples Federico II, 80131 Naples, Italy; 16 P.A.S.C.I.A. Center (Heart Failure Care Program, Childhood Heart Diseases and Those at Risk), University of Modena and Reggio Emilia, AOU Polyclinic of Modena, 41124 Modena, Italy; 17 Pediatric Pain and Palliative Care Service, IRCCS Istituto Giannina Gaslini, 16147 Genoa, Italy; 18 Community Medicine and Primary Care, AUSL Modena, 41124 Modena, Italy; 19 UOC Anesthesia and Intensive Care, IRCCS Istituto Giannina Gaslini, 16147 Genoa, Italy; 20 UOSID Trial Center, IRCCS Giannina Gaslini Institute, 16147 Genoa, Italy; 21 Scientific Directorate, IRCCS Istituto Giannina Gaslini, 16147 Genoa, Italy; 22 Pediatric Clinic, University of Ferrara, 44124 Ferrara, Italy; 23 Center for Metabolic Diseases and Atherosclerosis, University of Bologna, 40126 Bologna, Italy

**Keywords:** telemedicine, teleconsultation, telepediatrics, telemonitoring, televisit

## Abstract

Telemedicine is considered an excellent tool to support the daily and traditional practice of the health profession, especially when referring to the care and management of chronic patients. In a panorama in which chronic pathologies with childhood onset are constantly increasing and the improvement of treatments has allowed survival for them into adulthood, telemedicine and remote assistance are today considered effective and convenient solutions both for the chronic patient, who thus receives personalized and timely assistance, and for the doctors, who reduce the need for direct intervention, hospitalizations and consequent management costs. This Consensus document, written by the main Italian Scientific Societies involved in the use of telemedicine in pediatrics, has the objectives to propose an organizational model based on the relationships between the actors who participate in the provision of a telemedicine service aimed at minors with chronic pathologies, identifying specific project links between the areas of telemedicine in the developmental age from the first 1000 days of life to the age adult. The future scenario will have to be able to integrate digital innovation in order to offer the best care to patients and citizens. It will have to be able to provide the involvement of patients from the very beginning of the design of any care pathway, increasing where possible the proximity of the health service to citizens.

## 1. Introduction

Telemedicine is considered an excellent tool to support the daily and traditional practice of the health profession, especially when referring to the care and management of chronic patients [1,2,3]. The COVID-19 pandemic has brought to light all the fragility of health systems: the geographical and socio-economic disparities in accessing services and benefits, waiting times that are often too long to carry out examinations and specialist visits and the poor integration between the hospital system and medicine on the territory [4,5].

The ongoing evolution of the demographic dynamics and the consequent modification of the health needs of the population, with a growing proportion of patients with chronic pathologies, make it necessary to redesign the structure and organization of the network of services, above all with a view to strengthening the assistance area [6]. Technological innovation can contribute to a reorganization of health care, in particular by supporting the shift of the fulcrum of health care from the hospital to the community, through innovative care models centered on the citizen and facilitating access to services throughout the country [7,8]. The methods of providing health and social-health services enabled by telemedicine are fundamental in this sense, helping to ensure equity in access to care in remote areas, support for the management of chronic conditions, a channel of access to highly specialized, better continuity of care through multidisciplinary comparison and a fundamental aid for emergency–urgency services [9,10]. Moreover, telemedicine can improve patients’ quality of life through self-management solutions and remote monitoring, as well as for the purpose of an early dehospitalization [11,12].

In a panorama in which chronic pathologies with childhood onset are also constantly increasing, and the improvement of treatments has allowed survival for them into adulthood, telemedicine and remote assistance are today considered effective and convenient solutions both for the chronic patient, who thus receives personalized and timely assistance, and for the doctors, who reduce the need for direct intervention, hospitalizations and consequent management costs. There are many telemedicine initiatives at a national and international level, which too often, however, can be traced back to experiments, prototypes, projects and characterized by few cases [7,13,14]. In the face of this nonorganic diffusion of health services provided with telemedicine methods, it is necessary to have a shared governance model of telemedicine initiatives, which must have the central point in the specific knowledge of the health sector. A harmonization of the guidelines and application models of telemedicine is therefore necessary, as a prerequisite for the interoperability of services and as a requirement for the transition from an experimental logic to a structured logic of widespread use of telemedicine services.

This Consensus document is a narrative review written by the main Italian Scientific Societies involved in the use of telemedicine in pediatrics. It has the objectives to propose an organizational model based on the relationships between the actors who participate in the provision of a telemedicine service aimed at minors with chronic pathologies, identifying specific project links between the areas of telemedicine in the developmental age from the first 1000 days of life to the age adult. 

## 2. Methodology

The MEDLINE–PubMed database was searched from 2000 to 2022 to collect the literature. The following combinations of keywords were used: “telemedicine” AND “chronic disease” OR “asthma” OR “allergology” OR “cystic fibrosis” OR “cardiology” OR “type 1 diabetes” OR “neurology” OR “neuropsychiatry” OR “rheumatology” OR “rare disease” OR “palliative care” OR “first 1000 days of life” or “transitional medicine! AND “children” OR “paediatric” OR “pediatric” OR “adolescent”. We also performed a manual search of the reference lists of the obtained studies. Studies on telemedicine in chronic diseases and transition stages of life in childhood during the COVID-19 pandemic were included. 

## 3. Telemedicine and Chronicity

### 3.1. Allergology and Pulmonology

Digital health is of particular interest in the field of pediatric allergology and pulmonology in children, since allergic diseases and asthma in its various declinations are the most frequent chronic diseases in childhood and, at the same time, the pediatric patient, by definition, is more inclined to accept communication tools and educational messages based on play, digital technologies and innovation [15]. The American College of Allergy, Asthma and Immunology (ACAAI) already in 2017 produced a position paper on the use of telemedicine in allergic diseases, indicating its potential usefulness and main fields of application, but at the same time underlining areas of criticality, including those of certification, safe management of sensitive data and reimbursability of services [16]. In its turn, the European Academy of Allergy and Clinical Immunology (EAACI) published a policy paper on mobile health in 2020, aimed at indicating the usefulness of mobile technologies in the different allergic diseases of children and adults, as well as for a patient-centered approach based on precision medicine [17]. 

Overall, the use of telemedicine in allergology and pulmonology has numerous potentialities and unquestionable advantages, both for the patient and for the healthcare professional and the researcher themself [15,18,19]. These range from the possibility for the patient to acquire second opinions at a distance in the case of serious or complex pathologies, limiting travel costs and inconveniences, which is particularly important for fragile and socially disadvantaged patient populations, to the possibility for the clinician to assist the patient remotely, even in remote areas of the globe, using dedicated apps, electronic diaries, uni- or multiparameter monitors connected with control units for monitoring and transmitting data (e.g., SatO2, heart rate, respiratory rate and spirometry results). Last but not least, there is a clear advantage in offering more continuous home-based messages and educational tools for the patient and caregiver aimed at understanding the importance of adherence to therapy and avoidance of trigger factors. However, the importance of some crucial aspects for the possibility of applying such procedures in real life should not be overlooked, such as the need to digitally share sometimes very large amounts of data (medical records, imaging, etc.) and to have specific expertise, IT platforms and hardware tools suitable for the purpose [19]. 

Two different modes of telemedicine application can be distinguished, represented by the “synchronous” and the “asynchronous” modes. The first one takes place in real time and can be applied in direct mode or through facilitators, able to act as an interface between the patient and the physician [20,21]. The second takes place in deferred mode, through a two-way flow (from the patient to the health professional and vice versa) of information, using digital tools such as smartphones, tablets or PCs interfaced with portable medical devices (e.g., spirometers) or stationary (monitors connected to transmission centers), but also in the form of video recordings, tutorials and e-consulting useful for educational purposes for the patient and his or her family. All these means can help the pediatrician in improving home monitoring of the allergic patient or patient with chronic lung diseases and of the environment in which he or she lives (e.g., air quality, pollen counts), as well as in assessing the administration of therapy and the correct use of devices and in improving “home care” overall. 

Applied in particular to asthma, as to other pulmonological diseases, the applicability of telemedicine in the diagnostic phase clashes with the difficulty of carrying out a complete physical examination remotely, beyond a simple inspection through monitors and video cameras (e.g., evaluation of skin color and respiratory dynamics) and auscultation based on the perception of noises that can be heard at a distance (presence of wheezing, stridor, hoarseness, etc.) [21]. On the other hand, telemedicine may be easier to use when the clinical picture is limited to skin diseases (atopic dermatitis, urticaria and drug reactions), in which the web-based assessment of the type of lesion supplemented by the main anamnestic data (mode and time of onset or presence of itching) can alone allow a diagnosis. 

The world of digital technologies is, however, evolving, offering the possibility of teleauscultation, based on phonendoscopes capable of digitizing chest noises and transferring them to a PC, the applicability of which, however, in common clinical settings remains to be defined [8]. Of some interest are also some recently marketed portable devices that can be used in preschool wheezing, which can help the parent discriminate between wheezing and other respiratory noises [22]. At the same time, guidelines on asthma diagnosis place the presence of a clinical history, as well as a physical examination, suggestive of asthma at the center of the diagnostic pathway [23]. There is no doubt, therefore, that the correct anamnestic collection, which can easily be carried out remotely, can serve to exclude, or conversely confirm, with a few simple questions the suspicion of asthma at any age, including preschool (Table 1). Questions are based on the recommendations of the Global Strategy for Asthma Management and Prevention (GINA) [23].

Telemedicine, therefore, at least in the screening phase, can serve as an orientation tool for the clinician to direct the patient towards the most appropriate diagnostic pathway. Greater difficulties are encountered in the remote execution of respiratory function tests (spirometry, DLCO and measurement of respiratory resistance) and the assessment of bronchial inflammation (exhaled nitric oxide), tools of crucial importance in confirming the diagnosis of asthma [23]. These tests play a pivotal role in the diagnosis of asthma, along with all other obstructive and nonobstructive airway diseases (including bronchopulmonary dysplasia, interstitial disease and anatomical abnormalities of the conduction airways), but also in the assessment of the severity of pulmonary functional impairment and response to therapy. The literature demonstrates, in fact, the difficulty of meeting the acceptability and reproducibility criteria required by the guidelines to ensure the correct performance of spirometry in common outpatient settings in adults not adequately trained for the examination [24]. 

Of greater importance is telemedicine in the monitoring phase of asthma and other chronic lung diseases in patients with an established diagnosis. At this stage, spirometry is imaginable to play an important role in limiting hospital admissions in both emergency and outpatient settings, provided, however, that the patient has been adequately instructed in advance by the operator on the technique of performing the examination, is sufficiently cooperative and that family compliance is adequate. In this regard, experiences at the Brompton Hospital in London during the COVID-19 pandemic on about 400 children with asthma and other chronic lung diseases show that the implementation of spirometry at home in children accustomed to performing it is feasible and may serve to limit the use of hospital, so that its use will increase in the future [25]. Moreover, worth mentioning in this regard are experiences carried out in adults in countries with reduced economic resources, which show a good correlation between lung function tests performed with low-cost devices connected to a smartphone and spirometry acquired using more advanced, stationary spirometers [26]. 

Preliminary studies comparing the telemedicine approach, employing telepharmacilitators equipped with digital phonendoscopes and a high-resolution video camera, in the management of pediatric asthma have reported a noninferiority in disease control through remote management compared with the conventional in-presence approach [27]. There are also experiences of structuring a pediatric “virtual asthma clinic” using hybrid, in-presence and remote pathways, which demonstrate that the mixed approach can not only guarantee an equal or superior outcome in terms of symptom-free days and overall asthma control compared with the conventional approach but is even advantageous in terms of economic costs [28,29]. 

Additionally deserving of mention is meta-analysis work that has also reported that E-health educational interventions in adolescents and young adults improve self-management in asthma and other allergic diseases by optimizing inhalation techniques, increasing adherence and improving asthma control and patient quality of life [30,31]. 

### 3.2. Cystic Fibrosis

Cystic fibrosis (CF) is the most frequent genetic disease (autosomal recessive) in the Caucasian ethnic group. This disease is caused by a mutation in the CFTR (Cystic Fibrosis Transmembrane Regulator) gene, which is responsible for regulating the passage of chlorine in epithelial cells [32]. CF is a multiorgan disorder characterized by very dense secretions resulting in damage mainly to the lungs, where recurrent infections occur, and to the pancreas, which becomes progressively fibrotic, resulting in an inability to secrete the enzymes necessary for nutrient absorption. 

The care of people with CF is based on visits to the reference center every 3–4 months with monitoring of respiratory function, culture of sputum or pharyngeal swab, clinical examination and laboratory or, on some occasions, instrumental examinations. At each visit, the patient is generally followed-up by a multidisciplinary team that includes a pulmonologist, nurse, dietician, physiotherapist and psychologist [32]. The activation of a telemedicine system enables the patient to monitor certain biometric parameters such as oxygen saturation, weight and respiratory function at home and send them directly to the reference center. This means minimizing travel, increasing the patient’s quality of life, and also reducing the risk of cross-infection that can occur in the hospital environment. Other advantages of telemedicine reported in the literature are increased adherence to therapy [33], the possibility to follow the patient via video call in his or her respiratory physiotherapy and exercise practice [34,35] and early interception of flare-ups [36]. 

### 3.3. Cardiology

Pediatric cardiology, understood in this context as a set of congenital, syndromic or genetic heart diseases, represents a special field of cardiology and medicine. We refer in this context basically to patients who in the transition will become GUCH (Grown up Congenital Heart), today renamed ACHD (Adult Congenital Heart Disease) by the Scientific Societies [37]. In fact, this is a complex niche that is seeing the prevalence and incidence of affected newborns decrease, due to prevention and counseling, but is seeing the average life span increase due to advances in medical and interventional therapies. Increasingly efficient diagnostic and therapeutic pathways have led to a progressive and increasing reduction in mortality, resulting in an increasing population that requires and will require dedicated care and resources. The care of these patients requires specific and sectorial knowledge and experience, and the centers specialized in this field are limited and concentrated in a few reference sites, with consequent inconveniences both for the patients, who are forced to move and wait for long periods of time, and for the specialized structures, which are forced to carry heavy workloads [37]. 

In view of the above, pediatric cardiology represents an ideal context for telemedicine. There are now countless publications on the possible positive implications of using telemedicine to achieve predictive, participative, proactive, personalized and precision medicine [38]. 

To date, there are many telemedicine experiences in the field of pediatric cardiology, both at the national level (such as the "REMOTE" project in Sardinia, "Let’s get to everyone’s heart" in Tuscany, the Bambino Gesù Hospital project in Rome, the ADVICE project in the Lazio region, the GUCH Centre of the Gaslini Institute and ASL3 Liguria) and at the international level. With a view to optimizing telemedicine services in the field of pediatric cardiology, of great importance was the 2017 American Heart Association document [37]. Subsequently, the COVID-19 pandemic brought new insights on the topic and provided a sidereal boost to the use of telemedicine [39,40,41]. 

The potential of telemedicine in the field of pediatric cardiology encompasses all its expressions: teleassistance, teleconsultation, telemonitoring, tele-echography (from fetal monitoring to neonatal assessment), the follow-up of pediatric patients with known heart diseases, heart failure and electrophysiology, right up to the management of adult patients with congenital heart diseases [42,43]. It is necessary to structure organizational models in an appropriate and standardized manner in order to try to find the balance between health protection on the one hand and the optimization of healthcare resources on the other. 

### 3.4. Diabetology

Type 1 diabetes (DM1) management has evolved considerably in recent years. On the one hand, blood glucose sensors (CGMs) have almost completely replaced classical capillary blood glucose determination; on the other hand, multi-injection insulin therapy has also undergone a drastic downsizing, with the introduction of increasingly advanced pumps over the last two decades [44]. These devices (CGMs and pumps) belong to the class of medical technology devices and are constantly connected, via mobile phones, to cloud-based systems that allow real-time sharing of clinical data with one’s care team [45]. During the COVID-19 lockdowns, they were therefore resorted to and, in such a technologically advanced context, that the telemedicine solution has been initiated almost naturally in many pediatric diabetes centers. 

Since the lockdown began in March 2020, pediatric diabetes specialists had immediately perceived the need to continue to be a reference point for patients, who at that time were in danger of taking a back seat to the pandemic drama the country was experiencing. The regional referents of the Italian Society of Pediatric Endocrinology and Diabetology (SIEDP) immediately urged the local health authorities to consider validating the televisit tool as a substitute for the traditional in-person visit [46,47]. This tool has gradually been started in almost all Italian Pediatric Diabetes Centers. 

After an initial period in which the televisits at the IRCCS Gaslini Hospital in Genoa were carried out, upon the request of the individual patient and on unofficial systems, the Liguria Region included in the Single Regional Catalogue, the “Diabetes checkup televisit” service, effectively formalizing the visit not in the presence of the patient and allowing it to be reported. In the meantime, the Liguria Region has adopted a dedicated platform associated with the outpatient diaries, with which patient and doctor, in addition to being able to perform the televisit itself, can exchange documents such as reports and examinations. The patient, through the technological devices used to treat diabetes, downloads health data into the cloud in the days leading up to the visit in order to be able to share and comment on them during the visit itself. The doctor can then perform the service, possibly modifying the therapies and drawing up a report of the outpatient visit, digitally signed and shared in the regional platform. In fact, the process of the visit (downloading and sharing data) and the objectives of the visit (empowerment of the patient and family to achieve good glycemic control) are the same as those of the traditional visit. A survey was also carried out among patients and parents, concerning the satisfaction with the televisits performed [48]. From this survey, 90% of the respondents stated that they were very satisfied with the telemedicine service, that they had no problems communicating with the doctors and that they received adequate attention for their condition. In addition, 90% of patients who do not live in Genoa perceived a saving in time and money. Finally, 80% of the patients and parents who responded stated that they would continue to use the video call tool in the future [48]. Currently, since the pandemic emergency ended, more than 50% of outpatient services are carried out remotely at the Gaslini Diabetes Centre. The choice whether to carry out the visit in person or through telemedicine is almost always from the patient. However, at least once a year an in-person visit is performed for all patients followed. 

### 3.5. Neurology and Child Neuropsychiatry

Neurological and psychiatric disorders of pediatric age are often chronic and require long and complex follow-up and therapies that may last a lifetime. In many cases, an interdisciplinary approach is required involving inter- and intra-hospital interactions and hospital–territory integration. Telemedicine in this context assumes a strategic role in facilitating patient access to specialist care and the exchange of information between specialists [49]. 

The difficulty in accessing examinations and instrumental investigations during the lockdown period for the COVID-19 pandemic made it necessary to spread the use of televisiting and the telemonitoring of patients, accelerating a process already triggered by advances in communication technologies [50]. In Italy, it is estimated that more than 80% of epileptologists, neurologists and child neuropsychiatrists used remote systems during the pandemic to monitor their patients’ conditions and ensure proper therapeutic adherence [12,51]. 

The literature has shown that telemedicine is a suitable option in pediatric neurology and child neuropsychiatry, improves access to specialist expertise and increases satisfaction among patients and primary care providers [49,52]. Rametta et al. report 93% satisfaction among professionals and 86% among caregivers [52]. Libdeh et al. report that it was possible to provide telemedicine answers without recommending an in-patient visit in 56.9% of children with neurological pathology; the most common indications included paroxysmal episodes, headache and tremor [49]. For patients seen in the outpatient clinic, teleconsultations had reduced waiting times and facilitated the clinic visit. 

For the child with epilepsy, telemedicine can be of great help considering that the prevalence of the condition, the need for regular follow-up visits and the inadequate availability of medical specialists can make patient care difficult. Accurate anamnestic collection plays the main role, and neurological examination in the presence of the patient is rarely required; telemonitoring of clinical progress in relation to the efficacy of antiepileptic treatment of seizures is essential and can be performed effectively at a distance if parents have been well instructed in seizure recognition and appropriate diary keeping [53]. In addition, teleconsultation also plays a fundamental role [49]. At present, epilepsy centers of various levels are organizing themselves in order to be able to communicate effectively with each other in a network, according to the “Hub & Spoke” model. The network, therefore, becomes a tool to enable a multispecialist dialogue between the various reference figures, such as the epileptologist, the neuropediatrician, the attending physician, the nurse, the psychologist and the caregiver, but also to carry out consultations with both national and international experts. The teleconsultation (doctor–physician or doctor–technician) covers neuro-radiological diagnostic aspects, but especially neurophysiological ones, even in emergencies. Experience in pediatric, and even more so in neonatal, electroencephalography is not uniformly widespread throughout the country, and technological advances now allow teleconsultation even in the emergency–urgency and deferred emergency setting. In this context, a multicenter and multidisciplinary project has been coordinated by the Italian National Institute of Health, which has produced national guidelines for teleneurophysiology, with ample space for applications in pediatric and neonatal ages [12,51]. 

In the field of child psychiatry, the coexistence of chronicity and frailty, often not only limited to the child but also to the family unit, requires integrated interdisciplinary responses. Associated with this is the increasing number of patients, clearly favored by the social context induced by the pandemic [53]. Indeed, pathologies such as eating disorders, depression and social behavior disorders are becoming a real health emergency in school-age children and adolescents. Telemedicine can help address the widespread lack of access to specialist psychiatric care and psychological support [53]. 

Specialists in neurology and psychiatry consider telemedicine to be more useful in follow-up than initial assessment. The areas in which telemedicine would be less useful in an initial clinical evaluation, in neurology and child neuropsychiatry, are neuromuscular disorders, autoimmune disorders and autism [54]. Even in children under one year of age, telemedicine is considered inappropriate [55]. Today, hybrid systems are gradually being adopted for the management of children with neurological or neuropsychological illnesses, integrating in-patient visits (mainly in the initial diagnostic framing and when the neurological clinical examination requires an in-person visit to be decisive) with telemedicine monitoring. This involves clinical interviews, analysis of disease diaries (e.g., in the case of headache and epileptic seizures), sharing films of paroxysmal episodes and laboratory or instrumental test results [54,55]. In a recent study of patients with neuromuscular diseases, 80% reported lower stress levels thanks to telemedicine [55]. Furthermore, 100% of health professionals, 68% of parents and 59% of children stated that they would like a hybrid approach in the future. 

### 3.6. Rheumatology

In recent years, the usefulness of offering patients with chronic rheumatic diseases and their families an alternative to hospital checkup through the use of telemedicine has emerged in pediatric rheumatology [56]. This need arose from the frequent difficulty of ensuring constant and regular clinical monitoring of patients with active disease, in whom timely intervention may be necessary in the event of clinical flare-ups, side effects of drugs or difficulties in adhering to therapeutic prescriptions. In these cases, the optimal management of clinical follow-up may be hampered by the distance of the residence from the clinical Centre of reference, the financial commitment required by travel expenses, the child’s frequent school absences and the need for parents to abstain from work. 

The outbreak of the COVID-19 pandemic abruptly accelerated the consideration of telemedicine, as it forced pediatric rheumatologists to replace originally scheduled face-to-face visits with remote assessments [57,58,59]. Changing care needs have led to a rapid adaptation of the methodologies used in traditional clinical assessments to virtual reality. The new health and socio-economic scenario has, therefore, paved the way for the introduction of telemedicine and remote telemonitoring methods in diagnostics, health status assessment, therapeutic prescription and consultation with patients or local doctors [60]. In terms of research, this approach has introduced an innovative system in the management of data collection and pharmacovigilance registers. Studies are currently under way in various pediatric rheumatology centers to assess the reliability of telemedicine methods in care and research, including application in clinical trials. 

Although the use of telemedicine is encountering increasing interest and acceptance among clinicians, as well as patients and families, its generalized application is encountering some obstacles, not only of an institutional and legislative nature but also of a medical nature. Many pediatric rheumatologists have, in fact, expressed concern that telemedicine visits do not offer a sufficiently high standard of quality, as they make it difficult to adequately assess the patient’s clinical status [61]. In particular, they do not allow an accurate examination of the musculoskeletal system, precise recording of vital signs and effective communication with the patient. They also make the acquisition of laboratory data or imaging procedures problematic if this information needs to be obtained urgently [62]. Other limitations are inherent in the inability to provide adequate nursing or physiotherapy support during the visit. Added to this is the economic or technical difficulty for some families to have adequate tools (smartphones, PCs or tablets connected to the network) [63,64]. 

The advantages and obstacles associated with the application of telemedicine in pediatric rheumatology are listed in Table 2. 

### 3.7. Rare Diseases

Rare diseases are pathological conditions that are infrequent by definition but also poorly known, poorly studied and often lacking adequate treatment. The European Regulation on orphan medicinal products, also adopted by Italy, defines a rare disease when it affects less than 5 in 10,000 inhabitants [65]. Approximately 8000 different rare diseases are known, which together constitute a significant epidemiological and care burden. Although they often have clinical and care problems in common and justify their grouping, they require specialized and continuous care. Specialized centers capable of caring for these children are few and mainly located in third-level hospitals, but the need to circulate expertise while leaving the child in his or her territory of reference is increasingly recognized. For these reasons, telemedicine is a valuable resource for children with rare diseases [66,67]. 

Teleconsultation to the referring physicians in the child’s area of residence by centers with expertise in individual rare diseases makes it possible to select and optimize in-patient admissions. In combination with teleconsultations, this makes it possible on the one hand to reduce the workload for specialized facilities and, on the other hand, to reduce waiting times and the discomfort of transferring often fragile and multihandicapped children to centers far away from their territory of residence [66]. 

A major impetus for telemedicine applied to rare diseases was given by Europe with the establishment of European Reference Networks (ERNs) [68]. The 24 ERNs, officially established in March 2017, constitute “virtual hospitals” that provide a framework for the healthcare pathways of rare disease patients, integrating multispecialist expertise of excellence. In fact, the nodes of the network (Health Care Providers, HCP) of the different European countries including Italy have been selected for their offer of services to the patient and proven experience on specific diseases, documented by the number of cases followed. Their task is to facilitate discussion on individual patients with rare or complex diseases that require highly specialized care and concentrated knowledge and resources, in order to offer the best diagnostic possibilities without patients having to travel. The ERNs use a virtual system (CPMS—Clinical Patient Management System) of clinical patient care. Through this system, healthcare professionals from different Member States can connect to share their expertise, knowledge and experience related to specific disease cases [69]. ERNs are dedicated to patients with rare diseases of all ages and, given the epidemiological relevance of those with pediatric onset, the pediatric component plays an important role, in some cases (e.g., ERN-Eye) with some working groups dedicated to children’s specialists. 

### 3.8. Home Care and Palliative Care

Telemedicine applied to pediatric home palliative care aims to increase the patient’s quality of life by improving pain symptoms, strengthening monitoring and rationalizing the consumption of services offered (home and outpatient) [70]. The different objectives in the clinical and social field are to develop a new care mode of monitoring clinical data directly generated at the patient’s home (by the child themself, their family members and the equipment present at home); to allow effective and safe remote monitoring of the patient’s clinical condition through the use of the telephone, teleconferencing and remote management of the equipment present at home; to avoid unnecessary home visits, reducing the feeling of hospitalization while guaranteeing safety in the continuity of care, facilitating patients’ stay at home; reducing improper hospital admissions; allowing the patient and their family to remain at home and keep in touch with the outside world, both by encouraging school attendance (“school on line”) and by promoting neuropsychic abilities through play (Computer Game Therapy) and preserving the relationship with friends (especially for patients coming from other Italian regions or from abroad); and encouraging work continuity for parents whose occupation can take advantage of the possibility of tele-work [71]. 

Patients who may benefit from the use of telemedicine in this context are children and adolescents suffering from hemato-oncological diseases, patients with a need for adequate control of pain symptoms or other disturbing symptoms and patients who tend to be unable to access the specialist outpatient clinic or who are in an inactive phase of life. In these cases, it is possible to monitor at home general clinical data (heart rate, body temperature and O2 saturation, and in a second phase, the parameters coming from the respirator if the patient is subjected to home mechanical ventilation) where useful for a better calibration of therapy; frequency and intensity of pain (through direct pain assessment or indirectly through analysis of the number and frequency of morphine boluses delivered by the pump in case of intravenous–subcutaneous infusion) and presence and intensity of possible unforeseen and/or harmful adverse effects of opioid therapy; frequency and intensity of other disturbing symptoms (e.g., respiratory failure, restlessness, hallucinations, etc.); and possible malfunction of biomedical equipment at home. 

The data collected from the patient’s home can be sent via telematics, processed and managed through a specific computer platform that makes it possible to update the medical record in real time and create information flows of clinical data that will be transmitted in real time on specific media (smart phones) to healthcare workers. In the event of significant deviation of the parameters set from the normal range, emergency paths will be set up that set up automatic alarm systems that alert the staff responsible for care in real time. To achieve the above, it is necessary to prepare:

- A simple and immediate device that can generate an alert signal that is set up for prompt audio and video communication between patient–family and healthcare professionals;
- A system that generates information and therapeutic indications that are easy for patients and parents to understand (taking into account cultural and linguistic diversity) and that complies with medico-legal regulations;
- An adequate interface between the patient’s medical record (especially the indications in the “therapy” section) and the biomedical instrumentation present at home (infusion pumps—volumetric, syringe or prepared for patient-controlled analgesia—and home mechanical ventilators).

By implementing these pathways, one can realistically achieve a reduction in the problems of controlling the disturbing symptoms of these patients, optimizing their quality of life as much as possible, a reduction in the number of home visits by healthcare personnel, while maintaining an adequate continuity of care for the patient–family unit, and a reduction in hospital admissions.

## 4. Telemedicine before and beyond the Age of Development

### 4.1. Pregnancy and the First 1000 Days of Life

Despite weak signs of a recovery in the birth rate in 2021, the decline in births will not be halted [69]. Contributing to this decline is the situation of motherhood in search of a sustainable reconciliation between professional life and childcare needs [72]. In this context, the arrival of a child represents a delicate moment for the entire family unit. 

The first 1000 days of life (the period between conception and the first two years of a child’s life) are crucial for future health. Preventive, protective or curative interventions carried out promptly in this very first phase of life, in fact, lead to positive health results in the short, medium and long term, not only for the child and the adult who will be but also for the parents, the community and future generations. This first window of development of the child is characterized by the phase of construction of the organs, first of all of the brain and its functions, which in its plasticity is affected by exposure to a wide range of factors, positive or negative, which in many cases can be controlled. Furthermore, the development of the child’s cognitive and socio-relational skills depends a lot on the quality of their relationship with their parents and with their surrounding environment.

Expectant families and new parents benefit from prenatal courses and outpatient breastfeeding support offered from birth [73,74]. However, there is no uniformity of action among birth centers, and there is often a lack of integration with the support for the first months of life offered by local care services. Telemedicine can support pregnant women, new mothers, couples or families, both with more educational virtual meetings during pregnancy, and with a more practical approach in the post-partum period. By reaching families directly in their homes, physical barriers can be broken down and economic savings and less environmental impact can be achieved, not to mention the extreme practicality for a couple who are new parents in all cases that do not require in-person visits. At the same time, telemedicine allows for more effective and efficient integration between different care services. 

Three areas of solid applicability are as follows:

(1) Breastfeeding support → maternal exposure to the steps of the BFHI (Baby Friendly Hospital Initiative) [75], integrating telemedicine tools and resources in the area, offers solid support to the new mother and, by interacting directly within the home and with easy accessibility, provides a real contribution in support of the 10 steps to successful breastfeeding;
(2) Support for nutrition in pregnancy and the puerperium → The need for support for correct information on the role of maternal nutrition during lactation and during pregnancy is important [76,77,78]. Nutrition, in fact, is a crucial factor in reducing the incidence of and preventing chronic noncommunicable diseases, primarily obesity. This requires both early action in particular time windows, such as the introduction of complementary feeding, and a family environment made aware of the importance of a healthy routine and taste education [79,80]. In addition to this, specific counseling at mealtimes is possible during the introduction of complementary feeding, providing assistance precisely when needed, optimizing the pathway so that each parent–child pair can build together with the professional a carefully customized feeding pathway in light of the new scientific evidence on the subject, relating to a responsive approach to complementary feeding [81,82];
(3) Strengthening the parent–child dyad in terms of the interpretation of neurodevelopment-related signals → new parents feel the need to be able to decode their child’s behavioral signals, without this ability, especially for mothers, they feel incompetent and experience a feeling of deprivation that causes great anxiety and risks negatively influencing their relationship with their newborn.

It is important to support parents in carrying out their task and provide them with tools to be “effective parents”. Such initiatives support the humanization of care and support for families, and this correlates with favorable child outcomes. Therefore, a practical and effective new technology-based approach allows for the provision of personalized advice to parents according to their specific problems, simplifying and optimizing the dynamics of each family [83,84]. Table 3 summarizes the priorities for future development in this area. 

### 4.2. Transitional Medicine

There is an age at which one transitions from pediatric to adult care. This transition, which is the object of transitional medicine, must be "proposed, planned and scheduled from a child- and family-centered pediatric healthcare to a patient-centered and adult-oriented healthcare" [85,86,87,88]. Transition medicine is one of the challenges for Italian healthcare in which telemedicine can play a key role. The prevention and treatment of chronic pathologies can and must start in the pediatric age, and structured and formalized cultural, information and patient transfer pathways from pediatric to adult medicine are needed, or we would certainly lose a lot in terms of public health and health for our patients [88]. 

In the transition from pediatric to adult age, the amount of subjects receiving regular care is reduced by 75%. In addition, 20% of adolescents have some kind of chronic pathology; therefore, these subjects need resources, integration of knowledge and specific professionalism and, if not properly supported with a transition program, they may interrupt or suffer a certain degree of discontinuity in care, with the risk of serious repercussions on their health conditions. To this end, the national project “Transitional Care” aims to standardize the modalities of transition from pediatric to adult care in patients with chronic diseases and disabilities [89]. Telemedicine projects in this area focus on the need to standardize the collection, sharing and transfer of clinical information. The Electronic Health Record (Fascicolo Sanitario Elettronico) 2.0 alone cannot suffice, it is necessary to design and provide for data-driven and patient-driven pathways that are able to collect and contextualize data relating to each patient’s care pathway. Consequently, it appears necessary to foster the development of a shared digital platform between the actors in order to offer an adequate transition program and guarantee continuity of care. Moreover, one should not think of excluding families and patients from the design of such pathways and tools [90]. 

The pediatric patient who has become an adolescent enters adulthood. Therefore, their involvement appears indispensable to support the development of a progressive personal autonomy that is a stimulus to maintaining compliance and the development of a responsive “adulthood” for fruitful responsive parenting [91]. 

Last but not least, it is necessary not to limit one’s attention to chronic pathologies alone. The resurgence of sexually transmitted pathologies, psychological distress and the need to foster digital awareness during the growth of families and of the child and adolescent make it necessary to consider health budgets not mere pediatric tools but patient adaptive and indispensable tools in the care pathway from pediatric to adult age [92]. 

## 5. Conclusions

The development of tools for telemedicine allows to create new opportunities for the improvement of the health service through greater collaboration between the various professionals involved and the patients. The treatment of chronic diseases (Table 4), as well as pregnancy with the first 1000 days of life and transition from pediatric to adult care (Table 5), can represent a priority area for the application of telemedicine models. 

Telemonitoring can improve the quality of life of chronic patients through self-management solutions and remote monitoring, as well as for the purpose of early dehospitalization. In this way, it is possible to guarantee the creation of a network operating method which integrates the various institutional and noninstitutional actors responsible for taking charge of chronic conditions. On the other hand, the challenge of health systems in the coming years, linked to the high prevalence of chronic diseases even in childhood and to prolonged survival, which today allows children with chronic diseases to reach adulthood, must also be tackled through better use of the system, supported by information and communication technology. The introduction of telemedicine as an innovative organizational method has an immediate impact on making communication between the various actors usable and continuous and directing providers towards an appropriate use of resources, reducing the risks associated with complications, reducing the use of hospitalization, reducing waiting times and optimizing the use of available resources. The availability of timely and synchronous information also offers the possibility of measuring and evaluating healthcare processes with this organizational method through process and outcome indicators, including compliance with therapies.

There are many telemedicine initiatives at a national and international level, which too often, however, can be traced back to experiments, prototypes and projects, characterized by limited cases and high mortality of the initiative. In the face of this nonorganic diffusion of health services provided with telemedicine methods, it is necessary to have a shared governance model of telemedicine initiatives, which must have the central point in the specific knowledge of the health sector. A harmonization of the guidelines and application models of telemedicine is therefore necessary, as a prerequisite for the interoperability of telemedicine services and as a requirement for the transition from an experimental logic to a structured logic of widespread use of telemedicine services. This document, although not exhaustive, represents a desire to point out a starting point in a concrete and practical manner. From this perspective, authors from different backgrounds were involved in the drafting of this consensus, and the future possibility will be to involve other figures, primarily patients. It belongs, in fact, to the vision of connected care that the citizen/patient is engaged to actively participate in the care pathway in order to be assisted in a personalized, predictive and preventive manner. It is necessary to imagine and realize integrated, interoperable and fluid services, for which no barriers should be perceived between the different care settings. It is precisely for the realization of connected care that full interoperability of healthcare data is necessary. Barriers between hospital and territory, as well as between public and private, need to be overcome. It is necessary to stimulate the drafting of a global health pact that must be tightened, starting from the policies and translated to the decision making and operational levels according to a general data-driven One Health approach. The future scenario will have to be able to integrate digital innovation in order to offer the best care to the patient and the citizens. It will have to be able to provide the involvement of patients from the very beginning of the design of any care pathway, increasing where possible the proximity of the health service to citizens.

## Figures and Tables

**Table 1:** Useful questions for asthma diagnosis by teleconsultation in children and adolescents.

Preschool Age	Adolescence and Adulthood
Does mum or dad suffer from asthma?	Has the patient had or has atopic dermatitis?
Was the baby born at full term?	Does the patient often complain of nasal symptoms with serous rhinorrhea, sneezing and nasal itching, especially when exposed to allergens?
What was the baby’s weight at birth?	Does the patient experience symptoms of exertion intolerance (easy fatigability, coughing after minimal exertion, etc.)?
Did mum smoke during pregnancy?	Is the cough during flare-ups dry or oily?
Did the child presented respiratory distress at birth?	When the patient presents with bronchospasm, does the cough respond to bronchodilators?
Has the child had bronchiolitis? If yes, was he or she hospitalized?	Has the patient ever received background treatment with inhaled corticosteroids?
Does the child have or had atopic dermatitis?	In the last 4 weeks, how many times a week have you experienced daytime symptoms?

**Table 2:** The advantages and obstacles related to the application of telemedicine in pediatric rheumatology.

Advantages	Obstacles
Reduction in economic costs for families	Difficulty performing the musculoskeletal physical examination
Reducing patient absences from school	Inability to urgently perform laboratory tests or imaging studies
Reduction in parental absences from work	Lack of direct doctor/patient interaction
Facilitate the regular frequency of clinical checkups	Inability to provide nursing or physiotherapy support
Automatic collection of clinical and research data	Digital divide between families
Cost reduction for the National Health System	

**Table 3:** Telemedicine in pregnancy and in the first 1000 days: priorities for future development.

Pregnancy	Post-Partum
Training-type virtual meetings with specialized personnel: maternal nutrition, first care and minimal pathology of the newborn Specific nutritional counseling: importance of maternal nutrition during pregnancy	Specific counseling on breastfeeding, first care and minimal pathology of the newborn Specific nutritional counseling for mothers: the importance of maternal nutrition during breastfeeding Nutritional counseling in weaning Empowerment-oriented counseling on the interpretation of infant and child signals

**Table 4:** Telemedicine in children and adolescents with chronic disease.

Chronic Disease	Application	Limits
Pneumology and allergology	Screening, monitoring of asthma and other chronic lung diseases	Difficulty of carrying out a complete physical examination remotely, beyond a simple inspection through monitors and video cameras
Cystic fibrosis	Monitoring of oxygen saturation, weight, respiratory function and compliance to therapy	Inability to perform sputum cultures; difficulty in early diagnosis of respiratory exacerbations
Cardiology	Teleassistance, teleconsultation, telemonitoring and tele-ecography in patients with known heart diseases or heart failure	Absence of structured and standardized organizational models
Diabetology	Telemonitoring of health data and empowerment of the patient and family to achieve good glycaemic control	Absence of structured and standardized organizational models
Neurology and child neuropsychiatry	Monitoring of clinical symptoms and efficacy of antiepileptic treatment, teleconsultation (doctor–physician or doctor–technician) for neuroradiological diagnostic aspects, psychiatric and physiological consultation in emergency	Difficulty in initial assessment in patients with neuromuscular disorders, autoimmune disorders and autism and in children under one year of age
Rheumatology	Monitoring of patients with active disease in the event of clinical flare-ups, side effects of drugs and compliance to therapeutic prescriptions	Difficulty in adequately assessing the patient’s clinical status, lack of effective communication with the patient, difficulty in the acquisition of urgent laboratory data or imaging procedures and inability to provide adequate nursing or physiotherapy support
Rare diseases	Teleconsultation to the referring physicians in the child’s area of residence by centres with expertise	Absence of structured and standardized organizational models
Palliative care	Telemonitoring clinical data (heart rate, body temperature and oxygen saturation), pain symptoms, other disturbing symptoms (e.g., respiratory failure, restlessness, hallucinations, etc.) and possible malfunction of biomedical equipment at home in children and adolescents suffering from hemato-oncological diseases, in those with a need for adequate control of pain symptoms or other disturbing symptoms and in those who tend to be unable to access the specialist outpatient clinic or who are in an inactive phase of life	Absence of structured and standardized organizational models

**Table 5:** Telemedicine in children and adolescents in transition stages of life.

Transition Phase	Application	Limits
Pregnancy and the first 1000 days of life	Virtual meetings during pregnancy and with a more practical approach in the post-partum period. Breastfeeding support, support for nutrition in pregnancy and the puerperium, strengthening the parent–child dyad in terms of the interpretation of neurodevelopment-related signals	Absence of structured and standardized organizational models
Transition from pediatric to adult medicine	Sharing and transfer of clinical information	Absence of structured and standardized organizational models

## Data Availability

Not applicable.
